# Analysis of conditions affecting auto-phosphorylation of human kinases during expression in bacteria

**DOI:** 10.1016/j.pep.2011.09.012

**Published:** 2012-01

**Authors:** Amit Shrestha, Garth Hamilton, Eric O’Neill, Stefan Knapp, Jonathan M. Elkins

**Affiliations:** a Structural Genomics Consortium, Oxford University, Old Road Campus Research Building, Old Road Campus, Roosevelt Drive, Oxford OX3 7DQ, UK; b Gray Institute for Radiation Oncology and Biology, Old Road Campus Research Building, Old Road Campus, Roosevelt Drive, Oxford OX3 7DQ, UK

**Keywords:** Kinase, Expression, Purification, Auto-phosphorylation

## Abstract

Bacterial over-expression of kinases is often associated with high levels of auto-phosphorylation resulting in heterogeneous recombinant protein preparations or sometimes in insoluble protein. Here we present expression systems for nine kinases in *Escherichia coli* and, for the most heavily phosphorylated, the characterisation of factors affecting auto-phosphorylation. Experiments showed that the level of auto-phosphorylation was proportional to the rate of expression. Comparison of phosphorylation states following *in vitro* phosphorylation with phosphorylation states following expression in *E. coli* showed that the non-physiological ‘hyper-phosphorylation’ was occurring at sites that would require local unfolding to be accessible to a kinase active site. In contrast, auto-phosphorylation on unphosphorylated kinases that had been expressed in bacteria overexpressing λ-phosphatase was only observed on distinct exposed sites. Remarkably, the Ser/Thr kinase PLK4 auto-phosphorylated on a tyrosine residue (Tyr177) located in the activation segment. The results give support to a mechanism in which auto-phosphorylation occurs before or during protein folding. In addition, the expression systems and protocols presented will be a valuable resource to the research community.

## Highlights

► Auto-phosphorylation of over-expressed kinases is dependent on rate of expression. ► Evidence suggests hyper-phosphorylation happens during protein folding. ► Expression systems of nine human protein kinases are presented.

## Introduction

Kinases are extensively studied drug targets, but reagents for these investigations are not widely distributed or always affordable. In particular the availability of high-yielding validated bacterial expression systems for human protein kinases is limited. We present bacterial expression and protein purification methods for nine human protein kinases using the convenient hexahistidine tag method. The structures of all of these proteins have been determined and deposited in the protein data bank (PDB). However, in some cases there is no literature report of expression and purification protocols, and in some other cases the protein was produced with an alternative purification tag (thioredoxin or glutathione *S*-transferase (GST)).^1^ The kinases presented here are:

Mitogen-activated protein kinase 13 (MAPK13, also known as p38δ), which is a key molecule in MAPK signalling. Among the functions recently identified for p38δ specifically is a role in skin tumour development [1]. The structure of MAPK13 has been recently made available by SGX Pharmaceuticals Inc. (PDB ID: 3COI). The same group also released the structure of the kinase domain of polo-like kinase 4 (PLK4, also known as STK18) which is involved in centriole duplication [2] (PDB ID: 3COK) and the structure of serine/threonine kinase 24 (STK24, also known as MST3), a key enzyme in the regulation of cell death (PDB IDs: 3CKW, 3CKX). The structure of STK24 has also been determined using protein produced with a thioredoxin tag expression system at extra-low temperatures [3].

Mitogen-activated protein kinase 3 (MAPK3, also known as ERK1) is involved in regulation of meiosis and mitosis. The structure of MAPK3 has been determined, utilising a GST fusion construct expressed in *Escherichia coli* [4]. Mitogen-activated protein kinase 8 (MAPK8, also known as JNK) is involved in stress response. To crystallize the beta 1 isoform (NCBI Ref. NP_620634.1), Heo et al. expressed the protein as a C-terminal hexahistidine fusion in *E. coli* [5]. A selection of structures of MAPK8 isoform alpha 1 (NCBI Ref. NP_002741.1) in complex with different compounds have been published by Abbott Laboratories [6–9] using a C-terminal hexahistidine fusion (experimental details not published), and by GlaxoSmithKline, from protein produced as an N-terminal GST fusion in *E. coli* [10], expression methods also unpublished. We present expression of MAPK8 isoform alpha1 as an N-terminal hexahistidine fusion in *E. coli*.

Mitogen-activated protein kinase kinase 2 (MAP2K2, also known as MEK2), along with MAP2K1 (MEK1) is the upstream kinase of ERK. The structure of MAP2K2 has been determined (PDB ID: 1S9I), utilising a C-terminal hexahistidine fusion [11]. Oxidative-stress responsive 1 (OSR1, also known as OXSR1) is involved in regulation of Na^+^/K^+^/2Cl^−^ transporters. The structure of OSR1 has been determined using an N-terminal hexahistidine tag [12], as in our construct presented here. Mitogen-activated protein kinase 9 (MAPK9, also known as JNK2) is involved in stress response. The structure of MAPK9 has been determined by Roche (PDB ID: 3E7O), from bacterially expressed protein [13]. CHK2 checkpoint homologue (CHEK2) is involved regulation of the cell cycle following DNA damage. Various structures of CHEK2 have been determined, both with [14] and without the N-terminal FHA domain [15,16]. In each case the construct was also C-terminally truncated.

Here we present nine bacterial expression systems using hexahistidine tags for protein purification, including analysis of their phosphorylation states by mass spectroscopy. For the three most phosphorylated proteins, expression trials at different growth and induction conditions showed that heterogeneous auto-phosphorylation can be significantly influenced by expression protocols, while co-expression of λ-phosphatase yielded homogeneously non-phosphorylated protein. Our data suggest that phosphorylation events during expression can occur at non-physiological sites that are not accessible as substrate sites in folded proteins.

## Materials and methods

### Cloning

DNA for each of the proteins was amplified by PCR from template DNA obtained from a variety of sources. For MAPK13, MAPK3, PLK4, MAP2K2 and MAPK9, DNA was obtained from the Mammalian Gene Collection. The IMAGE Consortium Clone IDs for MAPK13, MAPK3, PLK4, MAP2K2 and MAPK9 are 2819932, 3634492, 5273226, 2961198 and 5528624, respectively. STK24 was obtained from synthetic DNA. MAPK8 and OSR1 were obtained from commercial sources, and CHEK2 DNA was generously donated by the National Institute for Medical Research, London, UK. PCR primers included appropriate 5′ extensions for subsequent incorporation into expression vectors.

The PCR products were incorporated into home-made expression vectors [17] by ligation-independent cloning as detailed in Table 1. All constructs contained either an N-terminal or C-terminal hexahistidine tag. All of the vectors used contained a TEV protease recognition site for removal of the tag except pNIC-CH where the hexa-histidine tag is non-cleavable. Full DNA sequences of all of the constructs and the translated protein sequences are available in the Supplementary material.

### Expression

The plasmids were transformed into *E. coli* BL21 (DE3) cells containing the pRARE2 plasmid from commercial Rosetta II (DE3) cells. The transformed cells were used to inoculate 10 ml of LB medium containing 34 μg/ml chloramphenicol and either 50 μg/ml kanamycin or 100 μg/ml ampicillin, and these cultures were grown overnight with shaking at 37 °C. The next day, the 10 ml culture was used to inoculate 1 l of LB medium containing either 40 μg/ml kanamycin or 80 μg/ml ampicillin in a 2 l baffled shaker flask. The cultures were grown with shaking at 37 °C until an OD_600_ of 0.50–0.68 was reached. The temperature was then reduced to 20 °C and protein expression was induced by addition of 0.5 mM isopropyl-β-d-thiogalactopyranoside. Cells were grown overnight before harvesting by centrifugation. Each cell pellet was resuspended in 20 ml binding buffer (50 mM Hepes pH 7.4, 500 mM NaCl, 5% glycerol, 5 mM imidazole, 0.5 mM *tris*(2-carboxyethyl)phosphine (TCEP), 0.2 mM phenylmethylsulfonyl fluoride (PMSF)) and lysed by sonication. The insoluble debris was removed by centrifugation.

### Protein purification

The target proteins were purified from the clarified cell extracts by immobilised metal ion chromatography (IMAC): Each cell extract was passed through 1 ml of Ni^2+^ resin in a gravity-flow column, and the resin was then washed with 20 ml of binding buffer, 10 ml of binding buffer containing 25 mM imidazole. Protein was eluted with 5 ml of binding buffer containing 250 mM imidazole. Each 5 ml eluted fraction was further purified on an S200 16/60 gel filtration column (GE Healthcare) pre-equilibrated in 20 mM Hepes pH 7.4, 500 mM NaCl, 0.5 mM TCEP. Gel filtration retention volumes are listed in Table 2. For the proteins with a TEV protease recognition site for tag cleavage, removal of the hexahistidine tag was accomplished by addition of TEV protease and incubation at 4 °C overnight.

### *In vitro* auto-phosphorylation

Protein samples were incubated for 1 h at room temperature with 1 mM ATP, 2 mM MgCl_2_ and 1 mM sodium orthovanadate. When MnCl_2_ was also added, the concentration was 1 mM.

### *In vitro* dephosphorylation

Protein samples were incubated overnight at room temperature with 1 mM MnCl_2_ and approximately 0.02 × molar ratio of λ-phosphatase (purified by GST-affinity from bacterial over-expression).

### Mass spectrometry

Intact mass measurements were acquired on an Agilent electrospray-ionisation time-of-flight (ESI-TOF) mass spectrometer attached to an Agilent liquid chromatography system using a C3 reverse-phase column. Proteins were separated from small molecules on the liquid chromatography system in 0.1% formic acid (FA) buffer, eluting with a methanol gradient, before injection into ESI-TOF.

For the phosphopeptide mapping, 5 μg of protein from each sample, diluted in 100 mM NH_4_HC0_3_, was reduced (10 mM dithiothreitol, 56 °C, 40 min) and alkylated (40 mM iodoacetamide, RT, 20 min) prior to overnight digestion at 37 °C with trypsin or chymotrypsin (Promega) at a final concentration of 5 μg/ml. The digest was stopped by reducing the pH to less than 3.0 with FA. Digested peptides were evaporated to dryness and resuspended in 2% trifluoroacetic acid (TFA), 70% acetonitrile (ACN). Phosphopeptides were enriched on titanium dioxide beads (10 μM titansphere, GL Sciences, Japan) pre-washed in 80% ACN, 2% TFA, 3 mg/ml 2,5-dihydroxybenzoic acid (DHB). The beads were washed with 2% ACN, 0.1% FA. Non-phosphopeptides were eluted with 80% ACN, 0.1% TFA, 300 mg/ml DHB before washing in 80% ACN 0.1% TFA. Phosphopeptides were eluted in 40% ACN, 15% aqueous NH_4_OH, evaporated to dryness and resuspended in 2% ACN, 0.1% FA. Phosphopeptides were analysed by online nanoflow liquid chromatography tandem mass spectrometry using a Dionex U300 (fitted with a Pepmap C18 column and eluted with a linear gradient of ACN) connected to a Bruker HCTultra ETD II ion trap through a nanoelectrospray ion source. The top four ions present in the survey scan were automatically selected for fragmentation by ETD. Alternatively ETD fragmentation was triggered by neutral loss of the phosphate group (loss of *m*/*z* 32.7, 38.7, 49.0, 58) in CID mode. Phosphopeptides were identified by Mascot (Matrix Science) searches of all tandem mass spectra against SwissProt.

## Results

### Construct design and expression analysis

**Fig. 1:**
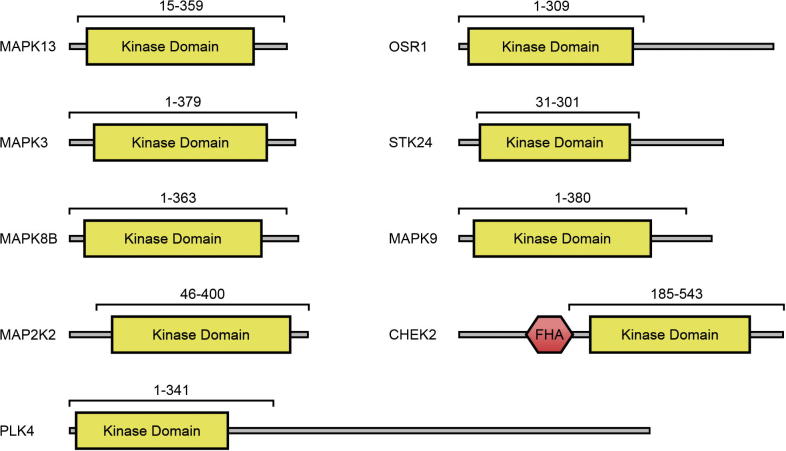
Domains present in each of the nine proteins in this study. For each protein the residue range covered by the expression construct in this study is illustrated above the domain diagram. Domain ranges were taken from analysis against the Pfam database [23].

For each target, a selection of bacterial expression constructs were designed, covering different ranges of the target kinase domain by using different N- and C-terminal truncations. These constructs were cloned into pET-based vectors carrying sequences for hexahistidine tags, transformed into an *E. coli* protein expression strain, and evaluated on a small scale for expression level of the target protein (results not shown). In some cases both N- and C-terminal hexahistidine tagged constructs were evaluated. A construct of each target that had among the highest yield of soluble protein expression is listed in Table 1 and illustrated in Fig. 1.

### Protein purification

**Fig. 2:**
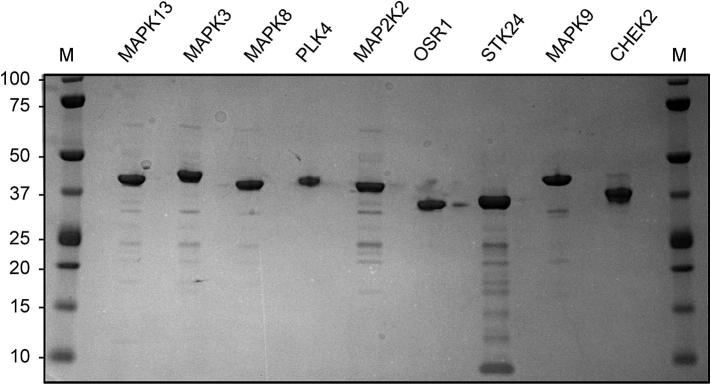
SDS–PAGE gel of protein samples following gel filtration chromatography. Standard molecular weight markers were Precision Plus from BioRad (M); the numbers in the vertical scale on the left show the mass in kDa. Each sample lane was loaded with 2 μg of protein from the pooled gel filtration chromatography fractions that had been boiled in the presence of sodium dodecyl sulphate. The gel was visualised by Coomassie blue staining.

To confirm the production of soluble protein and to evaluate the suitability of each construct for further work, each construct was expressed in 1 l of bacterial culture. Proteins were purified from the lysed cells by Ni^2+^ affinity chromatography followed by size-exclusion chromatography (Table 2). Equal amounts of each purified protein were run on a reducing, denaturing SDS–PAGE gel (Fig. 2), which showed all of the proteins migrating as an intact protein at the expected size. Only the STK24 gel sample showed the possibility of a limited amount of degradation or proteolysis (Fig. 2). The reported expression systems yielded 2.4–36 mg of pure recombinant protein per litre of culture medium.

### Protein characterisation

The size-exclusion chromatography showed that all of the proteins were monomeric in solution (Table 2), without significant levels of aggregation (chromatograms not shown). Electrospray (ESI) mass spectrometry analysis of the proteins was performed before and after removal of the hexahistidine tag (Tables 2 and 3). The results showed that all of the constructs expressed protein of the expected molecular weight, with the exception of MAPK9 and PLK4 where the difference in mass can be attributed to loss of an N-terminal methionine residue.

**Fig. 3:**
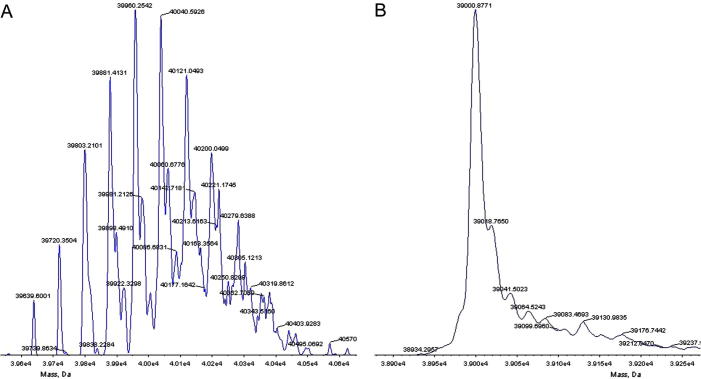
Example mass spectra of PLK4 following expression in *E. coli*. (A) Following expression without co-expressed λ-phosphatase. The peaks correspond to a range of phosphorylations from 8 to 16. (B) Following co-expression with λ-phosphatase. The peak corresponds to the molecular weight of the intact protein, minus an N-terminal methionine, with no phosphorylations.

Five proteins were phosphorylated (Tables 2 and 3), most notably PLK4 which showed a range of phosphorylation states up to 16 phosphorylations, depending on the experiment (Fig. 3). In the case of STK24, the difference of up to six phosphorylations with the hexa-histidine tag (which also contained linker residues and a TEV protease cleavage site), and only up to three without the tag, does not necessarily imply that the tag was triply phosphorylated, as a difference in ionisability between the two samples could potentially account for a difference in measurement sensitivity. Nevertheless, it is likely that at least some of the three removed phosphorylations were on the tag, whose sequence contains three serine and one threonine residues.

**Fig. 4:**
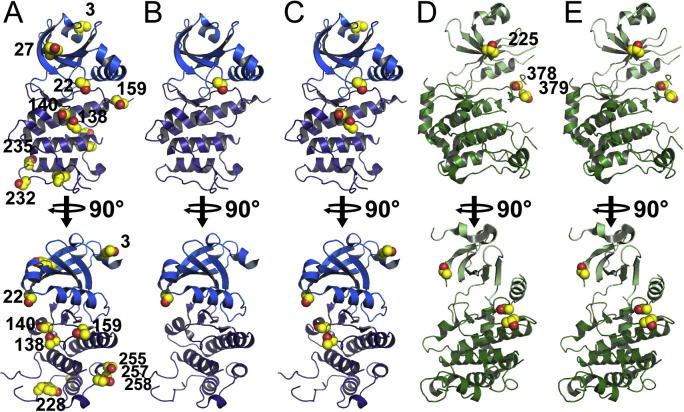
Phosphorylation sites of PLK4 and CHEK2 mapped onto the available crystal structures of these proteins (PDB IDs 3COK and 3I6 W). (A) Phosphorylation sites of PLK4 following expression in *E. coli*. (B) Phosphorylation sites of PLK4 following *in vitro* auto-phosphorylation. (C) Phosphorylation sites of PLK4 following *in vitro* de-phosphorylation of hyper-phosphorylated protein from *E. coli* expression. (D) Phosphorylation sites of CHEK2 following expression in *E. coli*. (E) Phosphorylation sites of CHEK2 following *in vitro* de-phosphorylation of hyper-phosphorylated protein from *E. coli* expression.

The phosphorylation sites for the two most heavily phosphorylated proteins, PLK4 and CHEK2, were mapped by proteolytic digestion followed by mass spectrometry (Tables 7 and 8). These sites were mapped onto the available crystal structures of these proteins (Fig. 4A and D). The sites were all on the surface of the protein, although many sites would clearly require local unfolding to bind to a kinase active site. Some of the sites could not be mapped onto the structures as they were on parts of the protein that were disordered in the structures, such as the kinase activation loop or the C-terminus of the protein.

### Dependence of phosphorylation level on experimental conditions

To investigate the hypothesis that the observed hyper-phosphorylation is non-physiological and is caused by the rapid over-expression of the kinases in a strong expression system, leading to phosphorylation of proteins before or during protein folding, experiments were designed to vary the rate of expression. PLK4, CHEK2 and STK24 were expressed with a low or high concentration of the induction agent IPTG, and with the induction taking place at 37 or 20 °C (Table 4).

The experiments showed that lower temperature at the time of induction reduced both the maximum number of phosphorylation sites and the number of phosphorylations in the most common phosphorylation state (modal phosphorylation state), while a reduction in the IPTG concentration also reduced both the maximum number and the mode. Therefore the differences observed were not solely due to differing enzyme efficiency with temperature. Additional experiments were performed without adding any IPTG; in all cases no protein was detectable on Coomasie-stained SDS–PAGE gels (data not shown), eliminating the possibility that the results were affected by ‘leaky’ expression of protein in the absence of inducer.

### *In vitro* auto-phosphorylation, following co-expression with λ-phosphatase

To test further the hypothesis that the observed hyper-phosphorylation during *E. coli* over-expression is non-physiological, *in vitro* auto-phosphorylation experiments were performed on PLK4, CHEK2 and STK24, starting with non-phosphorylated proteins. The constructs for PLK4, CHEK2 and STK24 were transformed into cells containing a λ-phosphatase expression plasmid. This co-plasmid also contained genes for the rare *E. coli* tRNAs. The expressions were repeated, and the phosphorylation state of the resultant proteins analysed by mass spectrometry. For all three proteins, co-expression with λ-phosphatase completely eliminated all phosphorylations (data not shown). Furthermore, in the case of STK24 the yield of soluble protein was substantially higher than when expressed without λ-phosphatase co-expression (data not shown).

These non-phosphorylated proteins were used for *in vitro* auto-phosphorylation experiments. The proteins were reacted with ATP and Mg^2+^, with or without the addition of Mn^2+^, for one hour at room temperature (equivalent to the 20 °C used for protein expression). Sodium vanadate was added to inactivate any trace amount of λ-phosphatase that might have remained in the purified protein. The result of the auto-phosphorylation experiments can be seen in Table 5. In each case, both the maximum number of phosphorylation sites and the modal value are reduced following *in vitro* phosphorylation, compared to following expression in *E. coli* in the absence of phosphatase.

For PLK4 these phosphorylation sites were also mapped by mass spectrometry (Table 8), which identified the location of three of the potential five positions. Two of the positions were on the kinase activation loop, including the activation residue Thr170, which is disordered in the available crystal structure, and one (Ser22) was on the glycine-rich loop of the kinase domain which is also generally flexible in the absence of a bound nucleotide or inhibitor (Fig. 4B).

### *In vitro* dephosphorylation, following *E. coli* expression auto-phosphorylation

The phosphorylated proteins produced by expression in *E. coli* without λ-phosphatase co-expression (Tables 2 and 3) were used for *in vitro* dephosphorylation experiments. The proteins were incubated with glutathione-*S*-transferase (GST) tagged λ-phosphatase in the presence of Mn^2+^. In each case the proteins remained phosphorylated, although with a reduction in the number of phosphorylated sites following the reaction (Table 6).

For CHEK2 and PLK4 these phosphorylation sites that were not removed by the λ-phosphatase treatment *in vitro* were again mapped by mass spectrometry (Tables 7 and 8, and Fig. 4C and E). The results showed that in each case the phosphatase removed all of the phosphorylations that were on the C-terminal lobe of the kinase domain while leaving most of the sites on the N-terminal lobe, including those on the activation loop.

## Discussion

Many kinases auto-phosphorylate during heterologous expression, and often an excessive number of phosphorylation states are observed (hyper-phosphorylation). This is a well-known observation and the additional phosphorylations can sometimes cause problems for subsequent applications of the purified proteins, for example if phosphorylations on a purification tag cause proteolytic tag removal to fail [18] or if phosphorylations occur on interfaces used to bind partner proteins. One hypothesis is that following induction, as the cell is being flooded with recombinant protein produced by a strong promoter, phosphorylations are introduced to the newly-translated protein during folding. In this way, phosphorylations are introduced to sites that are either not surface-exposed at all or, as observed here, that are otherwise non-reactive. These phosphorylations are sometimes not removed by subsequent *in vitro* phosphatase treatment, and cannot be replicated by *in vitro* auto-phosphorylation experiments.

The results on PLK4, CHEK2 and STK24 support this hypothesis, since a reduction in the rate of protein production (either through reduced inducer concentration or reduced temperature) lead to a reduction in the level of phosphorylation. The *in vitro* phosphorylation and dephosphorylation experiments also support this hypothesis since *in vitro* phosphorylation gave rise to significantly less phosphorylation in all cases as *E. coli* expression, and *in vitro* dephosphorylation (in the case of CHEK2) did not remove all of the phosphorylations introduced during *E. coli* expression.

The phosphorylation mapping on PLK4 and CHEK2 showed that for auto-phosphorylation, the large number of sites phosphorylated during expression in *E. coli* are located all over the surface of the protein, and many of these sites while not completely ‘buried’ in the protein interior would nevertheless require local unfolding to bind to a kinase active site. *In vitro*, however, only sites on the N-terminal lobe of the kinase were phosphorylated, although there were two unidentified sites which could have been located elsewhere. It is important to point out that the expressions were performed at similar temperatures to the auto-phosphorylation experiments (20 °C vs. room temperature) and so temperature-dependent flexibility was not a factor. In contrast, during *in vitro* dephosphorylation the sites that were removed were all on the C-terminal lobe, showing that the kinases and the λ-phosphatase have very different substrate recognition profiles. Considering that the N-terminal lobe of protein kinases is generally considered to more flexible than the C-terminal lobe, this contrast in behaviour of auto-phosphorylation compared to lambda-phosphatase specificity is interesting and could be connected in some way to known mechanisms of trans-activation of kinases, for example in both the cases of auto-phosphorylation or dephosphorylation the phosphorylation mapping showed that there are sites which are on the kinase domain itself and are accessible during *E. coli* expression, but inaccessible during the equivalent *in vitro* experiment.

While all of the proteins in this particular study could be produced in a stable, monomeric, form without significant aggregation despite the high levels of phosphorylation, in many cases proteins cannot be over-expressed in a soluble form except in the presence of a co-expressed phosphatase (e.g. YopH for expression of tyrosine kinases Fes [19] or Abl/Src [20]), and even when they are produced in a soluble form the yield may be higher with phosphatase co-expression as in the case of STK24 presented here. As discussed here and elsewhere, this is now a well-known method of producing soluble protein for protein kinases which exert problematic activity such as phosphorylation-associated toxicity when expressed in a heterologous system. The results presented here suggest that one reason for the success of phosphatase co-expression in such cases may be prevention of phosphorylation at sites that interfere with protein folding. There are other potential explanations of these observations such as co-expression of phosphatases reducing toxicity of the expressed target by removing undesirable phosphorylations that occur on essential endogenous proteins, and there may be combinations of mechanisms, but our results give additional support to the first hypothesis.

It is interesting to speculate on why some proteins remain soluble with extensive hyper-phosphorylation (such as PLK4 with up to 18 phosphorylations) while others cannot be produced in a soluble form in the absence of phosphatase. One simple reason would be the presence or absence of phosphorylation motifs on internal sites. However, the relative rates of protein folding and phosphorylation and the absence of eukaryotic chaperones such as hsp90 in bacteria could also be a factor; we have shown in this paper that the rate of expression affects the phosphorylation level and a protein that folds faster may be less susceptible to internal phosphorylation.

It is unlikely that phosphorylation sites that occur only during recombinant protein expression function as regulatory post-translational sites. However, it is interesting to note that a number of kinases auto-phosphorylate during folding either in a chaperonin-dependent or independent manner on sites that are not recognised by fully folded protein [21,22]. Specifically, the dual specificity kinases DYRK and GSK3 auto-activate on tyrosine residues. In our study, we also identified auto-phosphorylation on tyrosine residues for the kinase PLK4. Two of these tyrosines are located within the activation segment (Tyr169 and Tyr177) suggesting that PLK4 may share a similar activation mechanism as described for DYRK kinases. Strikingly Tyr177 was also observed in *in vitro* auto-phosphorylation experiments and similarly to DYRK kinases this tyrosine could not be dephosphorylated by phosphatase treatment.

Although in some cases co-expression with phosphatase is the only option, in others where kinases activate by auto-phosphorylation it may still be beneficial to express the protein in the presence of phosphatase, and subsequently perform the activation *in vitro* to avoid the introduction of non-physiological phosphorylations. The results presented here support this as an option that is available in principle although its use would of course depend on the particular protein of interest being suitable and on the type of study. For obtaining fully active kinases, in many cases an upstream kinase is required to provide the necessary phosphorylations.

In addition to the analysis of phosphorylation events, in this study we provided expression protocols for nine human protein kinases that have essential functions in cellular signalling. The reported expression systems were the most efficient ones from a larger number of constructs that have been cloned and tested in our laboratory. All of the constructs express soluble protein without the addition of large protein tags to enhance solubility such as glutathione-*S*-transferase. All of the proteins were purified by a simple two-step purification which yielded protein of sufficient purity for many types of experiment, and if required the expression and purification procedures could be optimised for higher yield, higher purity or larger scale. We hope therefore that these expression protocols will facilitate further biochemical studies in the signalling field that depends on efficient systems for the generation of stable recombinant proteins in economical bacterial host systems.

## Conflict of interest

None declared.

## Figures and Tables

**Table 1:** Expression constructs. Vector information has been recently published [17].

Target	GenBank ID	Residue Range	Vector	Purification Tag	Protease for tag cleavage	Antibiotic resistance
MAPK13	4506085	15–359	pLIC-SGC1	N-terminal His6	TEV	Ampicillin
MAPK3	38257141	1–379	pLIC-SGC1	N-terminal His6	TEV	Ampicillin
MAPK8	4506095	1–363	pLIC-SGC1	N-terminal His6	TEV	Ampicillin
PLK4	21361433	1–341	pNIC-CH	C-terminal His6	–	Kanamycin
MAP2K2	13489054	46–400	pNIC-CTHF	C-terminal His6	TEV	Kanamycin
OSR1	4826878	1–309	pNIC28-Bsa4	N-terminal His6	TEV	Kanamycin
STK24	20070158	31–301	pNIC28-Bsa4	N-terminal His6	TEV	Kanamycin
MAPK9	21237736	1–380	pNIC-CH	C-terminal His6	–	Kanamycin
CHEK2	6005850	185–543	pNIC28-Bsa4	N-terminal His6	TEV	Kanamycin

**Table 2:** Protein purification and mass spectrometry analysis. Gel filtration experiments were performed on four separate S200 16/60 columns and the observed retention volumes correspond to the molecular weights of the monomers. Deviations in retention volume are within the range expected for monomeric proteins of the molecular weights indicated. By comparison with molecular weight standards, a dimeric protein of 40 kDa MW would be expected to give a retention volume <75 ml under equivalent conditions. For the mass spectrometry analysis, the range is the minimum and maximum number of phosphorylations observed by mass spectrometry, and the modal value is the most highly populated phosphorylation state.

Target	Expected molecular mass (Da)	Gel filtration retention volume (ml)	Protein yield (mg/l culture)	Observed mass (Da)	Additional peaks	Interpretation/range of phosphorylations
MAPK13	42405.7	87	9.0	42408.0	None	Correct
MAPK3	45688.4	85	5.0	45690.9	1× Phosphorylation	Correct/range: 0–1 Modal value: 0
MAPK8	44464.5	85	14.7	44472.4	1× Phosphorylation	Correct/range: 0–1 Modal value: 0
PLK4	39130	95	27.0	39639.6	9–16× Phosphorylation	Loss of N-terminal Met/range: 8–16 Modal value: 13
MAP2K2	42515.7	80	2.4	42518.8	None	Correct
OSR1	37168.9	83	20.3	37171.1	None	Correct
STK24	33257.1	84	4.0	33339.4	2–6× Phosphorylation	Correct/range: 1–6 Modal value: 3
MAPK9	44615.5	80	36.7	44487.5	None	Loss of N-terminal Met
CHEK2	40835.1	85	25.8	40917.2	2–4× Phosphorylation	Correct/range: 1–4 Modal value: 2

**Table 3:** Mass spectrometry analysis of proteins after removal of the hexahistidine tag. The range is the minimum and maximum number of phosphorylations observed by mass spectrometry, and the modal value is the most highly populated phosphorylation state. The hexahistidine tag for PLK4 and MAPK9 was non-removable so these proteins do not feature in this table.

Target	Expected molecular mass (Da)	Observed mass (Da)	Additional peaks	Interpretation/range of phosphorylations
MAPK13	39940.1	39942.3	None	Correct
MAPK3	43222.8	43225.1	1× Phosphorylation	Correct/range: 0–1 Modal value: 0
MAPK8	41998.9	42001.6	1× Phosphorylation	Correct/range: 0–1 Modal value: 0
MAP2K2	40610.8	40613.6	None	Correct
OSR1	34703.3	34705.2	None	Correct
STK24	30791.5	30793.2	1–3× Phosphorylation	Correct/range: 0–3 Modal value: 1
CHEK2	38369.5	38451.2	2–4× Phosphorylation	Correct/range: 1–4 Modal value: 1

**Table 4:** Comparison of phosphorylation status under different expression conditions. The range is the minimum and maximum number of phosphorylations observed by mass spectrometry, and the modal value is the most highly populated phosphorylation state.

Temp. at induction [IPTG]	37 °C		20 °C	
0.05 mM	0.5 mM	0.05 mM	0.5 mM
PLK4	Range: 5–10	Range: 8–16⁎	Range: 3–9	Range: 4–9
Modal value: 8	Modal value: 13⁎	Modal value: 6	Modal value: 7

CHEK2	Range: 2–5	Range: 2–5	Range: 0–4	Range: 0–5
Modal value: 4	Modal value: 4	Modal value: 1	Modal value: 3

STK24	Range: 0–3	Range: 1–5	Range: 0–2	Range: 1–5
Modal value:1	Modal value: 2	Modal value: 0	Modal value: 2

⁎ Result from Table 2, the protein from the 50 ml scale expression under these conditions did not give a high quality mass spectrum.

**Table 5:** Phosphorylation state following *in vitro* auto-phosphorylation of protein co-expressed with λ-phosphatase. The range is the minimum and maximum number of phosphorylations observed by mass spectrometry, and the modal value is the most highly populated phosphorylation state.

	Auto-phosphorylation with ATP and Mg^2+^	Auto-phosphorylation with ATP, Mg^2+^ and Mn^2+^
PLK4	Range: 0–5	Range: 1–4
Modal value: 1	Modal value: 3

CHEK2	Range: 0	Range: 0
Modal value: 0	Modal value: 0

STK24	Range: 0	Range: 0
Modal value: 0	Modal value: 0

**Table 6:** Phosphorylation state following *in vitro* dephosphorylation with λ-phosphatase of protein expressed in *E. coli* without phosphatase co-expression. The range is the minimum and maximum number of phosphorylations observed by mass spectrometry, and the modal value is the most highly populated phosphorylation state.

	Dephosphorylation with λ-phosphatase
PLK4	Range: 0–6
Modal value: 1

CHEK2	Range: 0–2
Modal value: 0

STK24	Range: 1–4
Modal value: 3

**Table 7:** Locations of phosphorylation sites in CHEK2 as determined by proteolytic digestion followed by mass spectrometry. Underlined sites are in parts of the protein visible in the available structures.

CHEK2	Auto-phosphorylation in *E. coli*	Auto-phosphorylation *in vitro*	Remaining sites after dephosphorylation
Trypsin digest	*Thr225*		*Thr225*
Ser228		Ser228
Ser260		Ser260
*Thr378*		*Thr378*
*Ser379*		*Ser379*
Ser506		
Thr507		
Ser516		
Thr517		
Ser518		
Thr532		
Thr533		

Chymotrypsin digest	Ser260		
Thr272		
Thr383		

**Table 8:** Locations of phosphorylation sites in PLK4 as determined by proteolytic digestion followed by mass spectrometry. Underlined sites are in parts of the protein visible in the available structures.

PLK4	Auto-phosphorylation in *E. coli*	Auto-phosphorylation *in vitro*	Remaining sites after dephosphorylation
Trypsin digest	*Ser22*	*Ser22*	*Ser22*
*Tyr27*		
*Thr138*		*Thr138*
*Ser140*		*Ser140*
*Thr159*		
Tyr169		
Thr170	Thr170	
Thr174		Thr174
Tyr177	Tyr177	Tyr177
Ser179		Ser179
Thr184		
*Tyr228*		
*Ser232*		
*Ser235*		
*Ser255*		
*Ser257*		
*Ser258*		
Ser284		
Thr323		

Chymotrypsin digest	*Thr3*		*Thr3*
*Ser22*		
Thr184		
Ser186		

## Appendix A Supplementary data

Supplementary data 1 DNA and protein sequences of the constructs.

## Footnotes

1 *Abbreviations used:* GST, glutathione *S*-transferase; MAPK13, mitogen-activated protein kinase 13; STK24, serine/threonine kinase 24; MAPK3, mitogen-activated protein kinase 3; MAPK8, mitogen-activated protein kinase 8; MAP2K2, mitogen-activated protein kinase kinase 2; OSR1, oxidative-stress responsive 1; TCEP, *tris*(2-carboxyethyl)phosphine; PMSF, phenylmethylsulfonyl fluoride; IMAC, immobilised metal ion chromatography; ESI-TOF, electrospray-ionisation time-of-flight; FA, formic acid; TFA, trifluoroacetic acid; ACN, acetonitrile; DHB, 2,5-dihydroxybenzoic acid.
