# Supplementary material for: Analysis of conditions affecting auto-phosphorylation of human kinases during expression in bacteria
Source: Protein Expr Purif. 2012 Jan;81(1):136–43. doi: 10.1016/j.pep.2011.09.012 (PMC3445812; doi:10.1016/j.pep.2011.09.012)
Supplement: Supplementary data 1 — DNA and protein sequences of the constructs. [file mmc1.doc]

# Analysis of conditions affecting auto-phosphorylation of human kinases during expression in bacteria

Amit Shrestha^1^, Garth Hamilton^2^, Eric O’Neill^2^, Stefan Knapp^1^ and Jonathan M. Elkins^1(*)^

^1^ Structural Genomics Consortium, Oxford University, Old Road Campus Research Building, Old Road Campus, Roosevelt Drive, Oxford, OX3 7DQ, UK.

^2^ Gray Institute for Radiation Oncology and Biology, Old Road Campus Research Building, Old Road Campus, Roosevelt Drive, Oxford, OX3 7DQ, UK.

## Supplementary Material

### Contents:

Coding DNA sequences for the nine expression constructs. The protein sequences in red are removed by digestion with TEV protease.

## CHEK2

### Coding DNA Sequence

ATGCACCATCATCATCATCATTCTTCTGGTGTAGATCTGGGTACCGAGAACCTGTACTTCCAATCCATGACTGTAGATGATCAGTCAGTTTATCCTAAGGCATTAAGAGATGAATACATCATGTCAAAAACTCTTGGAAGTGGTGCCTGTGGAGAGGTAAAGCTGGCTTTCGAGAGGAAAACATGTAAGAAAGTAGCCATAAAGATCATCAGCAAAAGGAAGTTTGCTATTGGTTCAGCAAGAGAGGCAGACCCAGCTCTCAATGTTGAAACAGAAATAGAAATTTTGAAAAAGCTAAATCATCCTTGCATCATCAAGATTAAAAACTTTTTTGATGCAGAAGATTATTATATTGTTTTGGAATTGATGGAAGGGGGAGAGCTGTTTGACAAAGTGGTGGGGAATAAACGCCTGAAAGAAGCTACCTGCAAGCTCTATTTTTACCAGATGCTCTTGGCTGTGCAGTACCTTCATGAAAACGGTATTATACACCGTGACTTAAAGCCAGAGAATGTTTTACTGTCATCTCAAGAAGAGGACTGTCTTATAAAGATTACTGATTTTGGGCACTCCAAGATTTTGGGAGAGACCTCTCTCATGAGAACCTTATGTGGAACCCCCACCTACTTGGCGCCTGAAGTTCTTGTTTCTGTTGGGACTGCTGGGTATAACCGTGCTGTGGACTGCTGGAGTTTAGGAGTTATTCTTTTTATCTGCCTTAGTGGGTATCCACCTTTCTCTGAGCATAGGACTCAAGTGTCACTGAAGGATCAGATCACCAGTGGAAAATACAACTTCATTCCTGAAGTCTGGGCAGAAGTCTCAGAGAAAGCTCTGGACCTTGTCAAGAAGTTGTTGGTAGTGGATCCAAAGGCACGTTTTACGACAGAAGAAGCCTTAAGACACCCGTGGCTTCAGGATGAAGACATGAAGAGAAAGTTTCAAGATCTTCTGTCTGAGGAAAATGAATCCACAGCTCTACCCCAGGTTCTAGCCCAGCCTTCTACTAGTCGAAAGCGGCCCCGTGAAGGGGAAGCCGAGGGTGCCGAGACCACAAAGCGCCCAGCTGTGTGTGCTGCTGTGTTGTGA

### Translation

MHHHHHHSSGVDLGTENLYFQSMTVDDQSVYPKALRDEYIMSKTLGSGACGEVKLAFERKTCKKVAIKIISKRKFAIGSAREADPALNVETEIEILKKLNHPCIIKIKNFFDAEDYYIVLELMEGGELFDKVVGNKRLKEATCKLYFYQMLLAVQYLHENGIIHRDLKPENVLLSSQEEDCLIKITDFGHSKILGETSLMRTLCGTPTYLAPEVLVSVGTAGYNRAVDCWSLGVILFICLSGYPPFSEHRTQVSLKDQITSGKYNFIPEVWAEVSEKALDLVKKLLVVDPKARFTTEEALRHPWLQDEDMKRKFQDLLSEENESTALPQVLAQPSTSRKRPREGEAEGAETTKRPAVCAAVL

## MAP2K2

### Coding DNA Sequence

ATGCTTGACGAGCAGCAGAAGAAGCGGCTGGAAGCCTTTCTCACCCAGAAAGCCAAGGTCGGCGAACTCAAAGACGATGACTTCGAAAGGATCTCAGAGCTGGGCGCGGGCAACGGCGGGGTGGTCACCAAAGTCCAGCACAGACCCTCGGGCCTCATCATGGCCAGGAAGCTGATCCACCTTGAGATCAAGCCGGCCATCCGGAACCAGATCATCCGCGAGCTGCAGGTCCTGCACGAATGCAACTCGCCGTACATCGTGGGCTTCTACGGGGCCTTCTACAGTGACGGGGAGATCAGCATTTGCATGGAACACATGGACGGCGGCTCCCTGGACCAGGTGCTGAAAGAGGCCAAGAGGATTCCCGAGGAGATCCTGGGGAAAGTCAGCATCGCGGTTCTCCGGGGCTTGGCGTACCTCCGAGAGAAGCACCAGATCATGCACCGAGATGTGAAGCCCTCCAACATCCTCGTGAACTCTAGAGGGGAGATCAAGCTGTGTGACTTCGGGGTGAGCGGCCAGCTCATAGACTCCATGGCCAACTCCTTCGTGGGCACGCGCTCCTACATGGCTCCGGAGCGGTTGCAGGGCACACATTACTCGGTGCAGTCGGACATCTGGAGCATGGGCCTGTCCCTGGTGGAGCTGGCCGTCGGAAGGTACCCCATCCCCCCGCCCGACGCCAAAGAGCTGGAGGCCATCTTTGGCCGGCCCGTGGTCGACGGGGAAGAAGGAGAGCCTCACAGCATCTCGCCTCGGCCGAGGCCCCCCGGGCGCCCCGTCAGCGGTCACGGGATGGATAGCCGGCCTGCCATGGCCATCTTTGAACTCCTGGACTATATTGTGAACGAGCCACCTCCTAAGCTGCCCAACGGTGTGTTCACCCCCGACTTCCAGGAGTTTGTCAATAAATGCCTCATCAAGAACCCAGCGGAGCGGGCGGACCTGAAGATGCTCACAAACCACACCTTCATCAAGCGGTCCGAGGTGGAAGAAGTGGATTTTGCCGGCTGGTTGTGTAAAACCCTGCGGCTGAACCAGCCCGGCACACCCACGCGCACCGCCGTGGCAGAGAACCTCTACTTCCAATCGCACCATCATCACCACCATGATTACAAGGATGACGACGATAAGTGA

### Translation

MLDEQQKKRLEAFLTQKAKVGELKDDDFERISELGAGNGGVVTKVQHRPSGLIMARKLIHLEIKPAIRNQIIRELQVLHECNSPYIVGFYGAFYSDGEISICMEHMDGGSLDQVLKEAKRIPEEILGKVSIAVLRGLAYLREKHQIMHRDVKPSNILVNSRGEIKLCDFGVSGQLIDSMANSFVGTRSYMAPERLQGTHYSVQSDIWSMGLSLVELAVGRYPIPPPDAKELEAIFGRPVVDGEEGEPHSISPRPRPPGRPVSGHGMDSRPAMAIFELLDYIVNEPPPKLPNGVFTPDFQEFVNKCLIKNPAERADLKMLTNHTFIKRSEVEEVDFAGWLCKTLRLNQPGTPTRTAVAENLYFQSHHHHHHDYKDDDDK

## MAPK13

### Coding DNA Sequence

ATGCACCATCATCATCATCATTCTTCTGGTGTAGATCTGGGTACCGAGAACCTGTACTTCCAATCCATGAACAAGACCGCCTGGGAGCTGCCCAAGACCTACGTGTCCCCGACGCACGTCGGCAGCGGGGCCTATGGCTCCGTGTGCTCGGCCATCGACAAGCGGTCAGGGGAGAAGGTGGCCATCAAGAAGCTGAGCCGACCCTTTCAGTCCGAGATCTTCGCCAAGCGCGCCTACCGGGAGCTGCTGCTGCTGAAGCACATGCAGCATGAGAACGTCATTGGGCTCCTGGATGTCTTCACCCCAGCCTCCTCCCTGCGCAACTTCTATGACTTCTACCTGGTGATGCCCTTCATGCAGACGGATCTGCAGAAGATCATGGGGATGGAGTTCAGTGAGGAGAAGATCCAGTACCTGGTGTATCAGATGCTCAAAGGCCTTAAGTACATCCACTCTGCTGGGGTCGTGCACAGGGACCTGAAGCCAGGCAACCTGGCTGTGAATGAGGACTGTGAACTGAAGATTCTGGATTTTGGGCTGGCGCGACATGCAGACGCCGAGATGACTGGCTACGTGGTGACCCGCTGGTACCGAGCCCCCGAGGTGATCCTCAGCTGGATGCACTACAACCAGACAGTGGACATCTGGTCTGTGGGCTGTATCATGGCAGAGATGCTGACAGGGAAAACTCTGTTCAAGGGGAAAGATTACCTGGACCAGCTGACCCAGATCCTGAAAGTGACCGGGGTGCCTGGCACGGAGTTTGTGCAGAAGCTGAACGACAAAGCGGCCAAATCCTACATCCAGTCCCTGCCACAGACCCCCAGGAAGGATTTCACTCAGCTGTTCCCACGGGCCAGCCCCCAGGCTGCGGACCTGCTGGAGAAGATGCTGGAGCTAGACGTGGACAAGCGCCTGACGGCCGCGCAGGCCCTCACCCATCCCTTCTTTGAACCCTTCCGGGACCCTGAGGAAGAGACGGAGGCCCAGCAGCCGTTTGATGATTCCTTAGAACACGAGAAACTCACAGTGGATGAATGGAAGCAGCACATCTACAAGGAGATTGTGAACTTCAGCCCCATTGCCCGGAAGGACTCACGGCGCTGA

### Translation

MHHHHHHSSGVDLGTENLYFQSMNKTAWELPKTYVSPTHVGSGAYGSVCSAIDKRSGEKVAIKKLSRPFQSEIFAKRAYRELLLLKHMQHENVIGLLDVFTPASSLRNFYDFYLVMPFMQTDLQKIMGMEFSEEKIQYLVYQMLKGLKYIHSAGVVHRDLKPGNLAVNEDCELKILDFGLARHADAEMTGYVVTRWYRAPEVILSWMHYNQTVDIWSVGCIMAEMLTGKTLFKGKDYLDQLTQILKVTGVPGTEFVQKLNDKAAKSYIQSLPQTPRKDFTQLFPRASPQAADLLEKMLELDVDKRLTAAQALTHPFFEPFRDPEEETEAQQPFDDSLEHEKLTVDEWKQHIYKEIVNFSPIARKDSRR

## MAPK3

### Coding DNA Sequence

ATGCACCATCATCATCATCATTCTTCTGGTGTAGATCTGGGTACCGAGAACCTGTACTTCCAATCCATGGCGGCGGCGGCGGCTCAGGGGGGCGGGGGCGGGGAGCCCCGTAGAACCGAGGGGGTCGGCCCGGGGGTCCCGGGGGAGGTGGAGATGGTGAAGGGGCAGCCGTTCGACGTGGGCCCGCGCTACACGCAGTTGCAGTACATCGGCGAGGGCGCGTACGGCATGGTCAGCTCGGCCTATGACCACGTGCGCAAGACTCGCGTGGCCATCAAGAAGATCAGCCCCTTCGAACATCAGACCTACTGCCAGCGCACGCTCCGGGAGATCCAGATCCTGCTGCGCTTCCGCCATGAGAATGTCATCGGCATCCGAGACATTCTGCGGGCGTCCACCCTGGAAGCCATGAGAGATGTCTACATTGTGCAGGACCTGATGGAGACTGACCTGTACAAGTTGCTGAAAAGCCAGCAGCTGAGCAATGACCATATCTGCTACTTCCTCTACCAGATCCTGCGGGGCCTCAAGTACATCCACTCCGCCAACGTGCTCCACCGAGATCTAAAGCCCTCCAACCTGCTCATCAACACCACCTGCGACCTTAAGATTTGTGATTTCGGCCTGGCCCGGATTGCCGATCCTGAGCATGACCACACCGGCTTCCTGACGGAGTATGTGGCTACGCGCTGGTACCGGGCCCCAGAGATCATGCTGAACTCCAAGGGCTATACCAAGTCCATCGACATCTGGTCTGTGGGCTGCATTCTGGCTGAGATGCTCTCTAACCGGCCCATCTTCCCTGGCAAGCACTACCTGGATCAGCTCAACCACATTCTGGGCATCCTGGGCTCCCCATCCCAGGAGGACCTGAATTGTATCATCAACATGAAGGCCCGAAACTACCTACAGTCTCTGCCCTCCAAGACCAAGGTGGCTTGGGCCAAGCTTTTCCCCAAGTCAGACTCCAAAGCCCTTGACCTGCTGGACCGGATGTTAACCTTTAACCCCAATAAACGGATCACAGTGGAGGAAGCGCTGGCTCACCCCTACCTGGAGCAGTACTATGACCCGACGGATGAGCCAGTGGCCGAGGAGCCCTTCACCTTCGCCATGGAGCTGGATGACCTACCTAAGGAGCGGCTGAAGGAGCTCATCTTCCAGGAGACAGCACGCTTCCAGCCCGGAGTGCTGGAGGCCCCCTAG

### Translation

MHHHHHHSSGVDLGTENLYFQSMAAAAAQGGGGGEPRRTEGVGPGVPGEVEMVKGQPFDVGPRYTQLQYIGEGAYGMVSSAYDHVRKTRVAIKKISPFEHQTYCQRTLREIQILLRFRHENVIGIRDILRASTLEAMRDVYIVQDLMETDLYKLLKSQQLSNDHICYFLYQILRGLKYIHSANVLHRDLKPSNLLINTTCDLKICDFGLARIADPEHDHTGFLTEYVATRWYRAPEIMLNSKGYTKSIDIWSVGCILAEMLSNRPIFPGKHYLDQLNHILGILGSPSQEDLNCIINMKARNYLQSLPSKTKVAWAKLFPKSDSKALDLLDRMLTFNPNKRITVEEALAHPYLEQYYDPTDEPVAEEPFTFAMELDDLPKERLKELIFQETARFQPGVLEAP

## MAPK8

### Coding DNA Sequence

ATGCACCATCATCATCATCATTCTTCTGGTGTAGATCTGGGTACCGAGAACCTGTACTTCCAATCCATGAGCAGAAGCAAGCGTGACAACAATTTTTATAGTGTAGAGATTGGAGATTCTACATTCACAGTCCTGAAACGATATCAGAATTTAAAACCTATAGGCTCAGGAGCTCAAGGAATAGTATGCGCAGCTTATGATGCCATTCTTGAAAGAAATGTTGCAATCAAGAAGCTAAGCCGACCATTTCAGAATCAGACTCATGCCAAGCGGGCCTACAGAGAGCTAGTTCTTATGAAATGTGTTAATCACAAAAATATAATTGGCCTTTTGAATGTTTTCACACCACAGAAATCCCTAGAAGAATTTCAAGATGTTTACATAGTCATGGAGCTCATGGATGCAAATCTTTGCCAAGTGATTCAGATGGAGCTAGATCATGAAAGAATGTCCTACCTTCTCTATCAGATGCTGTGTGGAATCAAGCACCTTCATTCTGCTGGAATTATTCATCGGGACTTAAAGCCCAGTAATATAGTAGTAAAATCTGATTGCACTTTGAAGATTCTTGACTTCGGTCTGGCCAGGACTGCAGGAACGAGTTTTATGATGACGCCTTATGTAGTGACTCGCTACTACAGAGCACCCGAGGTCATCCTTGGCATGGGCTACAAGGAAAACGTGGATTTATGGTCTGTGGGGTGCATTATGGGAGAAATGGTTTGCCACAAAATCCTCTTTCCAGGAAGGGACTATATTGATCAGTGGAATAAAGTTATTGAACAGCTTGGAACACCATGTCCTGAATTCATGAAGAAACTGCAACCAACAGTAAGGACTTACGTTGAAAACAGACCTAAATATGCTGGATATAGCTTTGAGAAACTCTTCCCTGATGTCCTTTTCCCAGCTGACTCAGAACACAACAAACTTAAAGCCAGTCAGGCAAGGGATTTGTTATCCAAAATGCTGGTAATAGATGCATCTAAAAGGATCTCTGTAGATGAAGCTCTCCAACACCCGTACATCAATGTCTGGTATGATCCTTCTGAAGCAGAAGCTCCACCACCAAAGATCCCTGACAAGCAGTTAGATGAAAGGGAACACACAATAGAAGAGTGGAAAGAATTGATATATAAGGAAGTTATGGACTTGTGA

### Translation

MHHHHHHSSGVDLGTENLYFQSMSRSKRDNNFYSVEIGDSTFTVLKRYQNLKPIGSGAQGIVCAAYDAILERNVAIKKLSRPFQNQTHAKRAYRELVLMKCVNHKNIIGLLNVFTPQKSLEEFQDVYIVMELMDANLCQVIQMELDHERMSYLLYQMLCGIKHLHSAGIIHRDLKPSNIVVKSDCTLKILDFGLARTAGTSFMMTPYVVTRYYRAPEVILGMGYKENVDLWSVGCIMGEMVCHKILFPGRDYIDQWNKVIEQLGTPCPEFMKKLQPTVRTYVENRPKYAGYSFEKLFPDVLFPADSEHNKLKASQARDLLSKMLVIDASKRISVDEALQHPYINVWYDPSEAEAPPPKIPDKQLDEREHTIEEWKELIYKEVMDL

## MAPK9

### Coding DNA Sequence

ATGAGCGACAGTAAATGTGACAGTCAGTTTTATAGTGTGCAAGTGGCAGACTCAACCTTCACTGTCCTAAAACGTTACCAGCAGCTGAAACCAATTGGCTCTGGGGCCCAAGGGATTGTTTGTGCTGCATTTGATACAGTTCTTGGGATAAATGTTGCAGTCAAGAAACTAAGCCGTCCTTTTCAGAACCAAACTCATGCAAAGAGAGCTTATCGTGAACTTGTCCTCTTAAAATGTGTCAATCATAAAAATATAATTAGTTTGTTAAATGTGTTTACACCACAAAAAACTCTAGAAGAATTTCAAGATGTGTATTTGGTTATGGAATTAATGGATGCTAACTTATGTCAGGTTATTCACATGGAGCTGGATCATGAAAGAATGTCCTACCTTCTTTACCAGATGCTTTGTGGTATTAAACATCTGCATTCAGCTGGTATAATTCATAGAGATTTGAAGCCTAGCAACATTGTTGTGAAATCAGACTGCACCCTGAAGATCCTTGACTTTGGCCTGGCCCGGACAGCGTGCACTAACTTCATGATGACCCCTTACGTGGTGACACGGTACTACCGGGCGCCCGAAGTCATCCTGGGTATGGGCTACAAAGAGAACGTTGATATCTGGTCAGTGGGTTGCATCATGGGAGAGCTGGTGAAAGGTTGTGTGATATTCCAAGGCACTGACCATATTGATCAGTGGAATAAAGTTATTGAGCAGCTGGGAACACCATCAGCAGAGTTCATGAAGAAACTTCAGCCAACTGTGAGGAATTATGTCGAAAACAGACCAAAGTATCCTGGAATCAAATTTGAAGAACTCTTTCCAGATTGGATATTCCCATCAGAATCTGAGCGAGACAAAATAAAAACAAGTCAAGCCAGAGATCTGTTATCAAAAATGTTAGTGATTGATCCTGACAAGCGGATCTCTGTAGACGAAGCTCTGCGTCACCCATACATCACTGTTTGGTATGACCCCGCCGAAGCAGAAGCCCCACCACCTCAAATTTATGATGCCCAGTTGGAAGAAAGAGAACATGCAATTGAAGAATGGAAAGAGCTAATTTACAAAGAAGTCATGGATTGGGAAGAAAGAAGCAAGAATGGTGTTGTAAAAGATCAGCCTTCAGATGCAGCAGCGCACCATCATCACCACCATTGA

### Translation

MSDSKCDSQFYSVQVADSTFTVLKRYQQLKPIGSGAQGIVCAAFDTVLGINVAVKKLSRPFQNQTHAKRAYRELVLLKCVNHKNIISLLNVFTPQKTLEEFQDVYLVMELMDANLCQVIHMELDHERMSYLLYQMLCGIKHLHSAGIIHRDLKPSNIVVKSDCTLKILDFGLARTACTNFMMTPYVVTRYYRAPEVILGMGYKENVDIWSVGCIMGELVKGCVIFQGTDHIDQWNKVIEQLGTPSAEFMKKLQPTVRNYVENRPKYPGIKFEELFPDWIFPSESERDKIKTSQARDLLSKMLVIDPDKRISVDEALRHPYITVWYDPAEAEAPPPQIYDAQLEEREHAIEEWKELIYKEVMDWEERSKNGVVKDQPSDAAAHHHHHH

## OSR1

### Coding DNA Sequence

ATGCACCATCATCATCATCATTCTTCTGGTGTAGATCTGGGTACCGAGAACCTGTACTTCCAATCCATGTCCGAGGACTCGAGCGCCCTGCCCTGGTCCATCAACAGGGACGATTACGAGCTGCAGGAGGTGATCGGGAGTGGAGCAACTGCTGTAGTCCAAGCAGCTTATTGTGCCCCTAAAAAGGAGAAAGTGGCAATCAAACGGATAAACCTTGAGAAATGTCAAACTAGCATGGATGAACTCCTGAAAGAAATTCAAGCCATGAGTCAATGCCATCATCCTAATATTGTATCTTACTACACATCTTTTGTGGTAAAAGATGAGCTGTGGCTTGTCATGAAGCTGCTAAGTGGAGGTTCTGTTCTGGATATTATTAAGCACATTGTGGCAAAAGGGGAACACAAAAGTGGAGTCCTAGATGAATCTACCATTGCTACGATACTCCGAGAAGTACTGGAAGGGCTGGAATATCTGCATAAAAATGGACAGATCCACAGAGATGTGAAAGCTGGAAACATTCTTCTTGGAGAAGATGGCTCAGTACAGATTGCAGACTTTGGGGTTAGTGCTTTTTTAGCAACTGGTGGTGATATTACCCGAAATAAAGTGAGAAAGACCTTTGTTGGCACCCCTTGTTGGATGGCACCTGAAGTTATGGAACAGGTCCGTGGTTATGATTTCAAAGCTGATATTTGGAGTTTTGGAATTACAGCAATTGAATTGGCTACAGGGGCGGCTCCTTATCATAAATATCCACCAATGAAGGTTTTAATGCTGACACTGCAGAACGATCCTCCTTCTTTGGAAACTGGTGTTCAAGATAAAGAAATGCTGAAAAAATATGGAAAATCATTTAGAAAAATGATTTCATTGTGCCTTCAAAAAGATCCAGAAAAAAGACCAACAGCAGCAGAACTATTAAGGCACAAATTTTTCCAGAAAGCAAAGAATAAAGAATTTCTTCAAGAAAAAACATTGCAGAGAGCACCATGA

### Translation

MHHHHHHSSGVDLGTENLYFQSMSEDSSALPWSINRDDYELQEVIGSGATAVVQAAYCAPKKEKVAIKRINLEKCQTSMDELLKEIQAMSQCHHPNIVSYYTSFVVKDELWLVMKLLSGGSVLDIIKHIVAKGEHKSGVLDESTIATILREVLEGLEYLHKNGQIHRDVKAGNILLGEDGSVQIADFGVSAFLATGGDITRNKVRKTFVGTPCWMAPEVMEQVRGYDFKADIWSFGITAIELATGAAPYHKYPPMKVLMLTLQNDPPSLETGVQDKEMLKKYGKSFRKMISLCLQKDPEKRPTAAELLRHKFFQKAKNKEFLQEKTLQRAP

## PLK4

### Coding DNA Sequence

ATGGCGACCTGCATCGGGGAGAAGATCGAGGATTTTAAAGTTGGAAATCTGCTTGGTAAAGGATCATTTGCTGGTGTCTACAGAGCTGAGTCCATTCACACTGGTTTGGAAGTTGCAATCAAAATGATAGATAAGAAAGCCATGTACAAAGCAGGAATGGTACAGAGAGTCCAAAATGAGGTGAAAATACATTGCCAATTGAAACATCCTTCTATCTTGGAGCTTTATAACTATTTTGAAGATAGCAATTATGTGTATCTGGTATTAGAAATGTGCCATAATGGAGAAATGAACAGGTATCTAAAGAATAGAGTGAAACCCTTCTCAGAAAATGAAGCTCGACACTTCATGCACCAGATCATCACAGGGATGTTGTATCTTCATTCTCATGGTATACTACACCGGGACCTCACACTTTCTAACCTCCTACTGACTCGTAATATGAACATCAAGATTGCTGATTTTGGGCTGGCAACTCAACTGAAAATGCCACATGAAAAGCACTATACATTATGTGGAACTCCTAACTACATTTCACCAGAAATTGCCACTCGAAGTGCACATGGCCTTGAATCTGATGTTTGGTCCCTGGGCTGTATGTTTTATACATTACTTATCGGGAGACCACCCTTCGACACTGACACAGTCAAGAACACATTAAATAAAGTAGTATTGGCAGATTATGAAATGCCATCTTTTTTGTCAATAGAGGCCAAGGACCTTATTCACCAGTTACTTCGTAGAAATCCAGCAGATCGTTTAAGTCTGTCTTCAGTATTGGACCATCCTTTTATGTCCCGAAATTCTTCAACAAAAAGTAAAGATTTAGGAACTGTGGAAGACTCAATTGATAGTGGGCATGCCACAATTTCTACTGCAATTACAGCTTCTTCCAGTACCAGTATAAGTGGTAGTTTATTTGACAAAAGAAGACTTTTGATTGGTCAGCCACTCCCAAATAAAATGACTGTATTTCCAAAGAATAAAAGTTCAACTGATTTTTCTTCTTCAGGAGATGGAAACGCGCACCATCATCACCACCATTGA

### Translation

MATCIGEKIEDFKVGNLLGKGSFAGVYRAESIHTGLEVAIKMIDKKAMYKAGMVQRVQNEVKIHCQLKHPSILELYNYFEDSNYVYLVLEMCHNGEMNRYLKNRVKPFSENEARHFMHQIITGMLYLHSHGILHRDLTLSNLLLTRNMNIKIADFGLATQLKMPHEKHYTLCGTPNYISPEIATRSAHGLESDVWSLGCMFYTLLIGRPPFDTDTVKNTLNKVVLADYEMPSFLSIEAKDLIHQLLRRNPADRLSLSSVLDHPFMSRNSSTKSKDLGTVEDSIDSGHATISTAITASSSTSISGSLFDKRRLLIGQPLPNKMTVFPKNKSSTDFSSSGDGNAHHHHHH

## STK24

### Coding DNA Sequence

ATGCACCATCATCATCATCATTCTTCTGGTGTAGATCTGGGTACCGAGAACCTGTACTTCCAATCCATGGACCCAGAAGAGCTTTTTACAAAACTAGAGAAAATTGGGAAGGGCTCCTTTGGAGAGGTGTTCAAAGGCATTGACAATCGGACTCAGAAAGTGGTTGCCATAAAGATCATTGATCTGGAAGAAGCTGAAGATGAGATAGAGGACATTCAACAAGAAATCACAGTGCTGAGTCAGTGTGACAGTCCATATGTAACCAAATATTATGGATCCTATCTGAAGGATACAAAATTATGGATAATAATGGAATATCTTGGTGGAGGCTCCGCACTAGATCTATTAGAACCTGGCCCATTAGATGAAACCCAGATCGCTACTATATTAAGAGAAATACTGAAAGGACTCGATTATCTCCATTCGGAGAAGAAAATCCACAGAGACATTAAAGCGGCCAACGTCCTGCTGTCTGAGCATGGCGAGGTGAAGCTGGCGGACTTTGGCGTGGCTGGCCAGCTGACAGACACCCAGATCAAAAGGAACACCTTCGTGGGCACCCCATTCTGGATGGCACCCGAGGTCATCAAACAGTCGGCCTATGACTCGAAGGCAGACATCTGGTCCCTGGGCATAACAGCTATTGAACTTGCAAGAGGGGAACCACCTCATTCCGAGCTGCACCCCATGAAAGTTTTATTCCTCATTCCAAAGAACAACCCACCGACGTTGGAAGGAAACTACAGTAAACCCCTCAAGGAGTTTGTGGAGGCCTGTTTGAATAAGGAGCCGAGCTTTAGACCCACTGCTAAGGAGTTATTGAAGCACAAGTTTATACTACGCAATGCAAAGAAAACTTCCTACTTGACCGAGCTCATCGACTGA

### Translation

MHHHHHHSSGVDLGTENLYFQSMDPEELFTKLEKIGKGSFGEVFKGIDNRTQKVVAIKIIDLEEAEDEIEDIQQEITVLSQCDSPYVTKYYGSYLKDTKLWIIMEYLGGGSALDLLEPGPLDETQIATILREILKGLDYLHSEKKIHRDIKAANVLLSEHGEVKLADFGVAGQLTDTQIKRNTFVGTPFWMAPEVIKQSAYDSKADIWSLGITAIELARGEPPHSELHPMKVLFLIPKNNPPTLEGNYSKPLKEFVEACLNKEPSFRPTAKELLKHKFILRNAKKTSYLTELID
